# Mechanical Regulation of Nuclear Translocation in Migratory Neurons

**DOI:** 10.3389/fcell.2020.00150

**Published:** 2020-03-12

**Authors:** Naotaka Nakazawa, Mineko Kengaku

**Affiliations:** ^1^Institute for Integrated Cell-Material Sciences (WPI-iCeMS), Kyoto University Institute for Advanced Study, Kyoto University, Kyoto, Japan; ^2^Graduate School of Biostudies, Kyoto University, Kyoto, Japan

**Keywords:** neuronal migration, nuclear translocation, cytoskeleton, cellular mechanics, actomyosin, microtubule motors

## Abstract

Neuronal migration is a critical step during the formation of functional neural circuits in the brain. Newborn neurons need to move across long distances from the germinal zone to their individual sites of function; during their migration, they must often squeeze their large, stiff nuclei, against strong mechanical stresses, through narrow spaces in developing brain tissue. Recent studies have clarified how actomyosin and microtubule motors generate mechanical forces in specific subcellular compartments and synergistically drive nuclear translocation in neurons. On the other hand, the mechanical properties of the surrounding tissues also contribute to their function as an adhesive support for cytoskeletal force transmission, while they also serve as a physical barrier to nuclear translocation. In this review, we discuss recent studies on nuclear migration in developing neurons, from both cell and mechanobiological viewpoints.

## Introduction

Over a 100 years ago, Wilhelm His and Santiago Ramón y Cajal recognized that neurons were generated in specific germinal zones and migrated to their individual sites of function in the developing brain. Today researchers have caught up to their visionary studies and visualized neuronal migration through live imaging studies. Neuronal migration in earlier stages is critical for neuronal network formation in the later stages of brain development (Stiles and Jernigan, 2010; Silva et al., 2019). Disruption of neuronal migration thus causes brain malformations such as lissencephaly, which is accompanied by defects in neural network organization, manifesting as epilepsy, intellectual disability, and mental disorders. Previous studies have identified mutations in several genes encoding cytoskeletal motors and their associated molecules as the causes of these disorders (Manzini and Walsh, 2011; Cooper, 2013; Moon and Wynshaw-Boris, 2013; Buchsbaum and Cappello, 2019). These studies have expanded our knowledge of the roles of cellular signaling, including post-translational modifications of cytoskeletal molecules, in neuronal migration (Silva et al., 2018, 2019).

Migratory cells move long distances, frequently through confined spaces between other cells and extracellular matrices (ECMs) in tissues. Delivery of the nucleus, the largest and stiffest cargo, presents the biggest physical challenge for the cell to penetrate such confined environments. The nucleus is either pulled or pushed by the mechanical force generated by cytoskeletal motors, which are regulated by intracellular signals governing motor protein activity and cell polarity formation (Li and Gundersen, 2008; Fletcher and Mullins, 2010; Gundersen and Worman, 2013). In many migratory cells, the nucleus is harnessed to adhesions via actin cables and pulled forward by actomyosin contractility and integrin-mediated traction at the leading edge (Wolf et al., 2013; Wu et al., 2014). In contrast, other migratory cells including leucocytes in confined spaces require actomyosin contraction at the back in order to squeeze the nucleus through narrow pores (Lämmermann et al., 2008; Thomas et al., 2015). Rapid advances in mechanobiology have revealed how mechanical strains generated by physical confinement affect cytoskeletal dynamics in migrating cells (Stroka et al., 2014; Liu et al., 2015; Ruprecht et al., 2015).

However, our understanding of how the mechanical forces generated and sensed by cytoskeletal molecules drive nuclear translocation in migrating neurons lags behind that of other mesenchymal cells (Kengaku, 2018). In addition to intracellular signaling regulating cytoskeletal dynamics, one should consider the impact of mechanical properties of the nucleus, which is physically coupled to the cytoskeleton, in order to understand the mechanics of nuclear migration.

In this review, we summarize recent work on the role of mechanics in nuclear translocation in migrating neurons. First, we describe characteristic features of nuclear translocation and related nuclear machinery in neurons (see sections “Nuclear Migration in Neurons” and “The Machinery of the Nucleus: Its Mechanical Properties and Force Transmission”). Next, we illustrate how cytoskeletal motors drive nuclear translocation during neuronal migration in the brain with examples (see Section “Active Nuclear Translocation by Cytoskeletal Molecules in Neuronal Cells”). Finally, we discuss how the mechanical properties of the nucleus and extracellular environment impact nuclear translocation (see section “Cellular and Extracellular Mechanical Properties Affecting Nuclear Translocation in Neuronal Cells”).

## Nuclear Migration in Neurons

**FIGURE 1:**
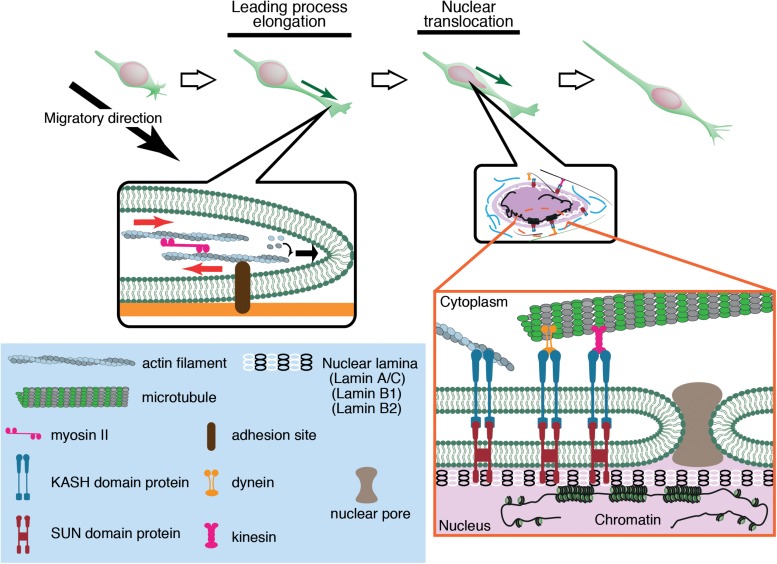
Neuronal migration is driven by leading process elongation and nuclear translocation independently. Leading process elongation is accompanied by growth cone extension driven by actin filament elongation and myosin II-dependent contraction. Nuclear translocation is driven by microtubule motor activities (dynein and kinesin) and actomyosin contraction.

Migrating neurons have a clear cell polarity and are subdivided into distinct compartments such as the growth cone, leading process, and cell body (Fletcher and Mullins, 2010; Cooper, 2013). These compartments are propelled independently by differential subsets of cytoskeletal systems, which coordinately regulate overall cell movement. Generally, neuronal migration is driven by two steps (Figure 1). The first step is the elongation of the leading process, where the plasma membrane at the growth cone is pushed by polymerizing *F*-actin. *F*-actin filaments are coupled by a molecular clutch to adhesions, thereby converting the myosin II-driven retrograde flow to a driving force for growth cone extension (Lin and Forscher, 1995; Lin et al., 1996; Bard et al., 2008; Lowery and Van Vactor, 2009; Kerstein et al., 2015). The second step is nuclear translocation, which is regulated differently from growth cone extension because of the physical separation of the nucleus from the growth cone. Differential regulation of these two steps causes a unique saltatory movement of the nucleus into the leading process (Edmondson and Hatten, 1987; Komuro and Rakic, 1995; Schaar and McConnell, 2005; Umeshima et al., 2007). This is in contrast to mesenchymal migration where the leading edge extension and nuclear translocation are regulated as sequential events.

## The Machinery of the Nucleus: Its Mechanical Properties and Force Transmission

The nucleus is the largest and stiffest cargo in migrating cells. The nucleus is demarcated by the nuclear envelope (NE), a double membrane barrier that separates the chromosomes from the cytoplasm. The inner nuclear membrane is underlined by the nuclear lamina, a meshwork of lamin intermediate filaments, which are critical for structural support of the nucleus (Aebi et al., 1986; Burke and Stewart, 2013; Gruenbaum and Foisner, 2015; Turgay et al., 2017; Ungricht and Kutay, 2017). Lamins are associated with the NE via the LINC (linker of nucleoskeleton and cytoskeleton) complexes formed by SUN (Sad1 and UNC-84) proteins and KASH (Klarsicht, ANC-1, and Syne Homology) proteins (Crisp et al., 2006; Sosa et al., 2013). SUN proteins are embedded in the inner nuclear membrane and bind to lamins in the nucleoplasm, whereas KASH proteins are in the NE lumen (Padmakumar et al., 2005; Sosa et al., 2013) and span the outer nuclear membrane, binding to actin and microtubule motors dynein and kinesin in the cytoplasm (Starr and Han, 2002; Zhen et al., 2002; Zhang et al., 2009). The nucleus inevitably receives significant mechanical strains during its active translocation into the tapering leading process. The driving forces for nuclear translocation are generated by actomyosin contraction and microtubule motor activity, which are transmitted to the NE via the LINC complex (Figure 1).

## Active Nuclear Translocation by Cytoskeletal Molecules in Neuronal Cells

### Actin-Myosin Based Nuclear Translocation

**FIGURE 2:**
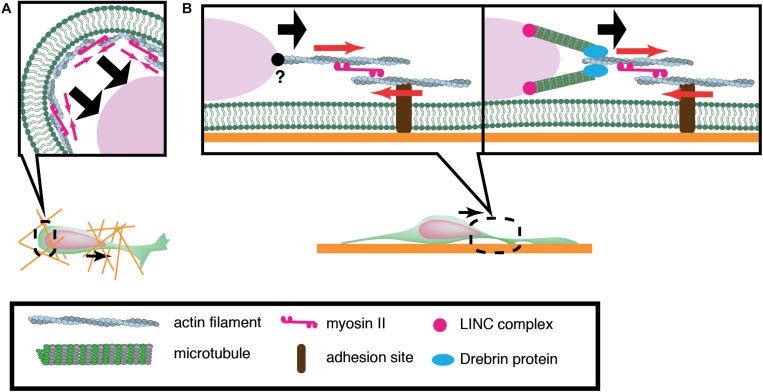
Schematic overview of actomyosin-based nuclear translocation in neurons. **(A)** Myosin II-dependent contraction of cortical actin (red arrows) at the rear of the nucleus might push the nucleus forward in a 3D environment. **(B)** On a 2D substrate, actomyosin contraction between the nucleus and adhesions (red arrows) in the leading process generates a traction force that pulls the nucleus. Direct interaction between actin filaments and the nucleus has not been confirmed in migratory neurons (Left). Actin filaments in the proximal leading process may transmit traction force to the nucleus via the perinuclear microtubules that are associated by microtubule-actomyosin coupling proteins such as drebrin (Right).

Of central importance to nuclear translocation are actomyosin-generated forces, yet previous studies have revealed a diversity in the sites of force generation, depending on cell types and assay systems. Granule cells and periglomerular cells, inhibitory interneurons in the olfactory bulb, arise in the anterior subventricular zone (SVZa) in the telencephalon and migrate rostrally to the olfactory bulb during development and throughout adult life. Dynamics of the rostral migration of SVZa neural precursor cells can be recapitulated in a culture of small SVZa explants embedded in a 3D Matrigel (Schaar and McConnell, 2005) (Figure 2A). The nucleus shows a characteristic saltatory movement toward a dilation formed in the leading process. Nuclear translocation is preceded by localization of non-muscle myosin IIB and membrane blebbing at the rear of the nucleus. Inhibition of myosin II activity at the nuclear rear by local application of blebbistatin suppresses nuclear translocation, suggesting that actomyosin generates a pushing force behind the nucleus. Accumulation of actomyosin at the nuclear rear is also observed during migration of cortical inhibitory interneurons from the medial ganglionic eminence (MGE) in the ventral telencephalon (Bellion et al., 2005; Martini and Valdeolmillos, 2010) (Figure 2A). Nuclear migration in MGE neurons is similarly inhibited by blebbistatin treatment, suggesting that myosin activation at the rear is critical for nuclear translocation. How does actomyosin contractility at the rear of the cell drive nuclear transport in these cells? Adhesion sites in the rear need to be detached for cell migration. A possible mechanism for detachment at the rear of the cell could be the mechanical disruption of integrin-ECM bonds by the actomyosin force behind the nucleus. Previous studies using keratocytes and cancer cell lines have demonstrated that actomyosin generates a traction force on focal adhesions which readily break integrin-ECM bonds (Jurchenko et al., 2014; Zhao et al., 2018). However, adhesions and stress fibers are less prominent in migrating neurons than in fibroblasts, seemingly downplaying the importance of de-adhesion at the cell posterior for neuronal migration (Jiang et al., 2015). Another possibility is that actomyosin contraction at the cell cortex constricts the cytoplasm at the rear and squeezes the nucleus to the front (Schaar and McConnell, 2005).

In contrast to the above mentioned inhibitory interneurons, dissociated cerebellar granule cells cultured on laminin-coated glass exhibit myosin IIB localization at the front of the nucleus prior to its saltatory movement (Solecki et al., 2009). Further studies using traction force microscopy (TFM) have demonstrated the generation of a force dipole at the site of myosin IIB accumulation in the leading process during neuronal migration (Jiang et al., 2015; Umeshima et al., 2019). These studies suggest that actomyosin is anchored to cell adhesions in the leading process and it exerts a pulling force to the nucleus in cerebellar granule cells cultured on a flat surface (Figure 2B). However, it remains elusive how the actomyosin force is transmitted to the nucleus during neuronal migration. It has been shown that F-actin is connected to the NE through the N-terminal CHD (Calponin Homology Domain) of nesprin-1/2 (Zhen et al., 2002; Padmakumar et al., 2004; Rajgor and Shanahan, 2013). Although the loss of nesprin-1 and 2 causes morphological defects in the mouse brain, an interaction between F-actin and nesprin-1/2 has not been detected in migrating neurons (Zhang et al., 2009). Recent studies have demonstrated that actomyosin in the leading process is anchored to perinuclear microtubules via an adaptor protein drebrin, rather than directly interacting with NE proteins (Trivedi et al., 2017).

The apparent diversity in actomyosin dynamics either in front or behind the nucleus has been attributed to differences among neuron types and/or diversity in migration substrates in different migration models (Trivedi and Solecki, 2011). Recent cell migration assays using rat and human mesenchymal cells and zebrafish germ layer progenitor cells have revealed that the subcellular localization of actomyosin in these cells is dramatically altered in 2D free surface and 3D confined space (Bergert et al., 2015; Liu et al., 2015; Ruprecht et al., 2015). Similarly, neuroepithelial cells alter the subcellular distribution of actomyosin and adopt different force mechanisms during nuclear translocation depending on cell shape and tissue morphology (Yanakieva et al., 2019). It is thus possible that neurons might also adopt differential cytoskeletal dynamics depending on the extracellular mechanical environment.

### Microtubule Based Nuclear Translocation

**FIGURE 3:**
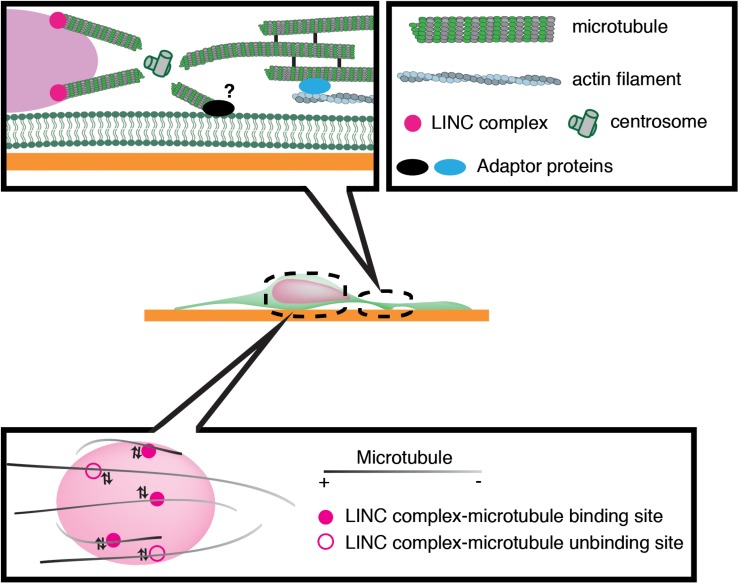
Schematic overview of microtubule-based nuclear translocation in neurons. The centrosome mostly locates in the front of the nucleus and emanates perinuclear microtubules. The centrosome may be anchored to the cell cortex via microtubule networks and actin filaments in the leading process or unknown membrane-associated adaptors. During neuronal migration, microtubules appear to repeat attachment and detachment to the nucleus via interactions of their associated motors with the LINC complex.

Previous studies have implicated microtubule motors as important regulators of nuclear translocation in the developing brain. Migrating neurons are polarized along the direction of migration, with the centrosome typically positioned in front of the nucleus. During neuronal migration, the centrosome and Golgi apparatus first move to a distal dilation that emerges in the leading process, and the nucleus then translocates toward the centrosome in the dilation (Bellion et al., 2005; Nishimura et al., 2017). Here, microtubules are thought to uniformly orient their plus-ends to the nucleus, harnessing the NE to the centrosome via the LINC complex. It is widely accepted that the nucleus is pulled forward to the centrosome by the minus-end-directed motor activity of cytoplasmic dynein, as the inhibition of dynein or its regulator LIS1 attenuates nuclear displacement (Hirotsune et al., 1998; Shu et al., 2004; Tanaka et al., 2004; Tsai et al., 2007). In this scenario, the centrosome has to be anchored to the cell cortex of the leading process in order to generate a traction force against the cell membrane or ECM, which pulls the nucleus forward (Aumais et al., 2001; De Simone et al., 2018). However, previous live imaging studies have revealed dynamic movement of the centrosome around the nucleus, raising doubts about whether the centrosome is tightly associated to the cell cortex (Umeshima et al., 2007; Wu et al., 2018) (Figure 3). More recent studies using cerebellar granule cells have suggested that the actin- and microtubule tip-binding protein drebrin links the perinuclear microtubules to F-actin in the leading process and mediates a strong actomyosin traction force at the integrin-ECM bonds to the nucleus (Ong and Solecki, 2017; Trivedi et al., 2017) (Figure 2). Other studies have indicated that perinuclear microtubules are associated with non-centrosomal microtubules in the leading process, which may be anchored to actomyosin and/or the cell cortex (Rao et al., 2016).

We have recently demonstrated that the nucleus undergoes frequent rotation during migration of cerebellar granule cells (Wu et al., 2018). Live-cell imaging suggests that microtubules around the nucleus are of mixed polarity and dynamically attach to and detach from the nucleus (Figure 3). This evidence supports the idea that kinesin and dynein motors exert transient forces to multiple small points on the NE, and thereby induce forward translocation when the net force acts on the center, which otherwise generates torque and drives rotation (Wu and Kengaku, 2018).

**FIGURE 4:**
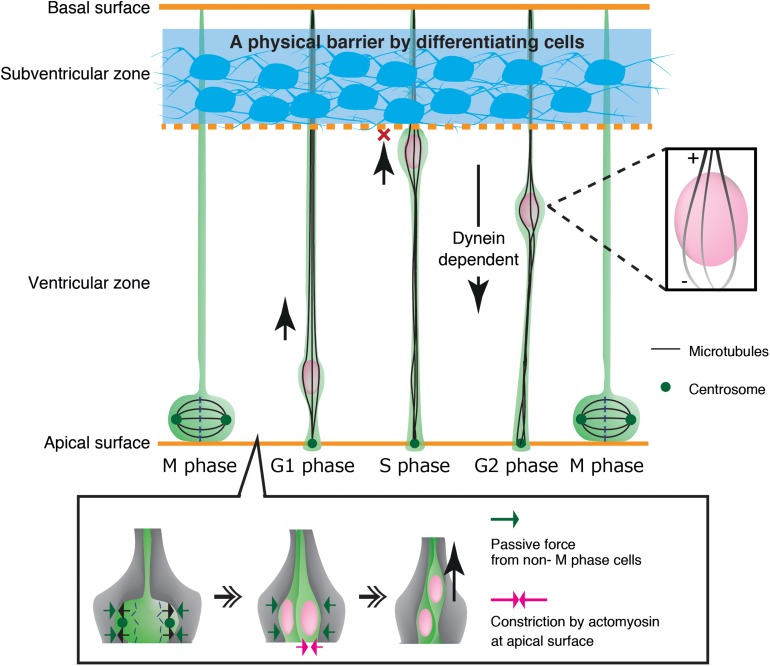
Mechanical regulation of nuclear translocation in neuroepithelial cells. Differentiating cells in the subventricular zone work as a physical fence which prevents invasion of the S phase nuclei in the upper ventricular zone (**Top**, blue region). On the apical surface, lateral expansion of mitotic cells is suppressed by the reactive force of surrounding non-M phase cells. Apical constriction by actomyosin further assists basal translocation of the dividing nuclei **(Bottom)**.

As another example, neuroepithelial cells in the developing neural tube show cyclic nuclear translocation between the apical and basal surface of the ventricular zone (VZ) in concert with the cell cycle, in a process known as interkinetic nuclear migration (Figure 4) (Spear and Erickson, 2012b; Lee and Norden, 2013; Miyata et al., 2015). The centrosome is anchored to the apical endfoot and emanates microtubules along the cell longitudinal axis with their plus-ends toward the basal side (Spear and Erickson, 2012a). Thus, dynein motor activity drives nuclear translocation from the basal side to the centrosome, which is anchored to the apical surface (Tsai et al., 2007; Hu et al., 2013). In contrast, the plus-end-motor activity of KIF1A has been implicated in nuclear translocation from the apical to basal surface away from the centrosome, although the anchor point at the basal side remains unclear (Tsai et al., 2007). An alternative mechanism for apical-to-basal migration involves actomyosin behind the nucleus, constricting the plasma membrane around the apical surface. In this case, the nucleus is squeezed toward the basal side, similarly to SVZa neurons (Norden et al., 2009; Schenk et al., 2009). Thus, synergistic transactions between microtubules and actomyosin are important for the generation and transmission of the force driving nuclear translocation in various contexts.

## Cellular and Extracellular Mechanical Properties Affecting Nuclear Translocation in Neuronal Cells

The nucleus is exposed to high shear stress from the surrounding tissue during migration in confined interstitial spaces. Besides cytoskeletal forces generated within the cell, the mechanical properties of the nucleus and extracellular microenvironment are important determinants of nuclear translocation.

### Nuclear Stiffness and Lamins

The nucleus shows extensive deformation when it passes through constrictions in the interstitial spaces in brain tissue (Wu et al., 2018). Accordingly, the nucleus should have an optimal viscoelastic property that allows flexible deformation and tolerance against shear stress during migration. A critical determinant of nuclear stiffness is the set of type V intermediate filament proteins, called lamins, which underlie the inner nuclear membrane. Lamins are composed of four major isoforms: lamins A and C (products of the LMNA gene by alternative splicing), lamin B1 (encoded by LMNB1) and lamin B2 (encoded by LMNB2). The stoichiometry of lamin A/C and lamin B1/B2 determines the viscoelastic properties of the NE and provides mechanical strength to the nucleus against various physical strains from the cytoplasm and extracellular environment (Swift et al., 2013; Harada et al., 2014).

Lamin A/C levels vary greatly among cell types, with strong correlations to tissue stiffness (Swift et al., 2013). Importantly, the laminA/C level is determined by both transcriptional and post-transcriptional regulation. Transcription of lamin A/C is activated by a transcription factor RARG that is translocated to the nucleus when cells are cultured on stiff substrate. The level of lamin A/C transcripts is thus low in soft tissues, including the brain (Swift et al., 2013). Additionally, lamin A/C proteins are phosphorylated at specific sites, and are thereby subjected to proteolysis under low mechanical stress in soft tissues (Buxboim et al., 2014). It has also been shown that lamin A abundance in the brain is further reduced by a brain-specific micro RNA miR-9 which specifically suppresses the 3′ UTR of the lamin A splice form (Jung et al., 2012, 2013). Generally, lamin A expression in migratory cells, such as immune cells and metastatic cancer cells, is lower than in adherent cells (Shin et al., 2013; Matsumoto et al., 2015; Irianto et al., 2016). Migrating neurons in the developing brain also express very low levels of lamin A/C (Coffinier et al., 2011). Nuclei containing low levels of lamin A are softer, and might be adept at deforming and passing through tiny interstitial spaces during migration.

A very low abundance of lamin A/C has been observed in embryonic cells, and LMNA knockout mice show little or no pathology during development, although postnatal mice develop cardiomyopathy or muscular dystrophy (Sullivan et al., 1999). In contrast, genetic ablation of B-type lamins causes abnormal neuronal migration and malformation of the brain cortex (Coffinier et al., 2011; Young et al., 2012, 2014). In experiments that specifically depleted lamin B1 in the forebrain of mice, the nucleus of migrating cortical neurons showed blebbing and/or fragmentation at the nuclear front (Jung et al., 2013). On the other hand, lamin B2 ablation causes abnormal elongation of the front of the nucleus in migratory neurons (Coffinier et al., 2010). These observations suggest that loss of B-type lamins impairs nuclear lamina integrity and tolerance to cytoskeletal pulling forces during neuronal migration.

It has been shown that the mechanical properties of the nucleus are also affected by the positioning and structure of chromatin (Pajerowski et al., 2007; Mazumder et al., 2008; Furusawa et al., 2015; Shimamoto et al., 2017). In most eukaryotic nuclei, heterochromatin regions primarily associate with nuclear lamina via lamins, forming lamina associated domains (LAD) which contribute to nuclear stiffness. In neuronal cells with low expression of lamin A, the inner nuclear membrane protein lamin B receptor plays important roles in LAD formation (Clowney et al., 2012; Solovei et al., 2013). Thus, lamin B receptor might have complementary roles with lamin A in neurons.

Nuclear lamins might have an important role for protection of the genomic architecture and DNA from mechanical stress. Recent studies have shown that knockdown of lamin A increases nuclear rupture and DNA damage in cancer cell lines (Xia et al., 2018). Similarly, mechanical stress in confined spaces induces excessive nuclear deformation and nuclear rupture, followed by DNA repair responses in bone marrow derived dendritic cells and cancer cell lines with low lamin A expression (Denais et al., 2016; Raab et al., 2016). In contrast, it has never been reported that young neurons with very low lamin A are susceptible to nuclear rupture during migration (Chen et al., 2019). Instead, disruption of lamin B1or lamin B2 has been shown to cause nuclear rupture and precocious cell death in migrating neurons, supporting that B-type lamins are more critical for brain development (Jung et al., 2013; Chen et al., 2019). It is unknown how the nuclear envelope with low lamin A maintains structural integrity, but other lamins such as lamin B and lamin B receptor may possibly substitute for lamin A in organizing the nuclear lamina structure in young neurons. Further studies are required to understand the relationships of lamin subtypes and their precise contributions to nuclear stiffness and durability.

### Mechanical Properties of the Extracellular Environment

Mechanical properties of surrounding tissues also contribute to interkinetic nuclear migration in neuroepithelial cells in the ventricular zone (Miyata et al., 2015). These cells undergo mitosis that causes cell crowding and increased pressure at the apical surface, contributing to pushing the nucleus up to the basal side (Kosodo et al., 2011; Okamoto et al., 2013). Further studies have demonstrated that the apical surface of the ventricular zone undergoes actomyosin-dependent contraction, further crowding the apical surface with elastic processes of surrounding progenitor cells. These dense processes exert a centripetal force on the dividing nuclei, thereby enhancing their dorsal displacement (Shinoda et al., 2018) (Figure 4). Recent studies have also demonstrated that apical-to-basal migration stops at the boundary of the ventricular zone and the SVZ by the physical fence made by differentiated SVZ neurons (Watanabe et al., 2018) (Figure 4).

The ECM is a scaffolding architecture for cells, which binds to integrins and promotes the formation of adhesions, thereby regulating various cellular functions including migration (Hynes, 2009; Bonnans et al., 2014). It has been shown that the mechanical properties of the ECM affect integrin signaling in migrating fibroblasts, so that cell migration is directed toward more rigid substrates in a process known as durotaxis (Choquet et al., 1997; Wang et al., 2002; Plotnikov et al., 2012; Sunyer et al., 2016). In developing neurons, growth cone extension also depends strongly on local mechanical properties (Kostic et al., 2007; Suter and Miller, 2011; Koser et al., 2016). However, whether and how substrate stiffness might affect nuclear translocation is not well understood.

Mechanical stresses to the nucleus influence the interior genome architecture and may affect cellular responses, including gene expression (Uhler and Shivashankar, 2017). It has been demonstrated that the rigidity of the extracellular environment is sensed and transmitted to biochemical signals that induce cytoskeletal remodeling and cytoplasmic-to-nuclear translocation of transcription factors including YAP/TAZ, and thereby affect cell differentiation, morphology, and survival (Engler et al., 2006; Dupont et al., 2011; Nakazawa et al., 2016; Smith et al., 2018; Wolfenson et al., 2018; Yang et al., 2019). Recent studies have also demonstrated that brain stiffness exhibits sharp gradients across layers and regions, which may affect multiple steps of neuronal differentiation including cell fate determination and circuit pathfinding (Iwashita et al., 2014; Koser et al., 2016; Barnes et al., 2017). In fact, the culture substrate with the stiffness of living brain tissue promotes production of dorsal cortical neurons from neural stem cells, suggesting a deep impact of mechanical properties of extracellular environment on gene expression in differentiating neurons (Iwashita et al., 2019). It remains of great interest to clarify if mechanical properties of surrounding tissues affect genome architecture and/or gene expression during neuronal migration.

## Conclusion

In this review, we summarized recent studies on the mechanical regulation of nuclear translocation in neurons. It is clear that the nucleus is translocated by both actomyosin traction forces and microtubule motors walking along rigid microtubule cables. However, it remains unclear how these cytoskeletal elements are stabilized on the substrate (anchor points) to transmit the force to the nucleus (application points). The research field is awaiting further improvements of emerging techniques for quantification of small mechanical forces in soft and complex tissues (Campàs et al., 2014; Roca-Cusachs et al., 2017; Mongera et al., 2018). For instance, a nesprin tension biosensor is a promising new tool for the quantification of the local, transient force applied to the nucleus in live cells (Arsenovic et al., 2016).

Additionally, increasing evidence highlights the need to carefully consider the impact of mechanical properties of the nucleus and the extracellular environment, and perhaps those of the cytoskeleton and the cell membrane, on nuclear translocation in 3D confined tissue. Recent studies using leukocytes and cancer cell lines suggest that the nucleus serves as a mechanical guide to choose paths of a proper width during confined space migration (Lautscham et al., 2015; Renkawitz et al., 2019). Manipulation of the mechanical properties of the extracellular environment in 3D tissue is required to demonstrate the physiological significance of these findings *in vitro*. A combination of organoid culture and mechanobiology is becoming a powerful system to overcome some of the limitations of *in vivo* experiments (Okuda et al., 2018; Garreta et al., 2019). Similarly, further improvement of micro/nanofabrication techniques to design 3D patterned substrates with various mechanical properties is needed to study the interplay between migrating cells and the surrounding tissue.

DNA damages by mechanical stress during confined migration may cause heterogeneity in cancer cells. It has been shown that nuclear deformation by confined migration induces prolonged DNA breaks due to mislocalization of DNA repair factors in the cytosol, which causes accumulation of chromosomal aberrations (Irianto et al., 2017). Extrapolating from this notion, an interesting hypothesis is that the nuclear deformation under mechanical stress during confined migration might influence the genome architecture and gene expression, and thereby affect the final fate and destination of migrating neurons. Emerging techniques for visualization of genome architecture in live cells in combination with high throughput genome sequencing analyses will offer greater opportunities to answer these questions (Miyanari et al., 2013; Natale et al., 2017; Conic et al., 2018).

## Author Contributions

All authors listed have made a substantial, direct and intellectual contribution to the work, and approved it for publication.

## Conflict of Interest

The authors declare that the research was conducted in the absence of any commercial or financial relationships that could be construed as a potential conflict of interest.
